# Decreased Hospital Length of Stay for ICH and PE after Adoption of an Artificial Intelligence-Augmented Radiological Worklist Triage System

**DOI:** 10.1155/2022/2141839

**Published:** 2022-08-18

**Authors:** Michael Petry, Charlotte Lansky, Yosef Chodakiewitz, Marcel Maya, Barry Pressman

**Affiliations:** ^1^Cedars-Sinai Medical Center, 8705 Gracie Allen Dr, Los Angeles 90048, CA, USA; ^2^Stanford University School of Medicine, 300 Pasteur Drive, Stanford 94305-5105, CA, USA

## Abstract

The purpose of the study was to determine whether there was a difference in the length of stay (LOS) for inpatients diagnosed with intracranial hemorrhage (ICH) or pulmonary embolism (PE) prior to and following implementation of an (AI) triage software. A retrospective review was performed for patients that underwent CT imaging procedures related to ICH and PE from April 2016 to October 2019. All patient encounters that included noncontrast head computed tomography (CT) or CT chest angiogram (CTCA) procedures, identified by the DICOM study descriptions, from April 2016 to April 2019 were included for ICH and PE, respectively. All patients that were diagnosed with ICH or PE were identified using ICD9 and ICD10 codes. Three separate control groups were defined as follows: (i) all remaining patients that underwent the designated imaging studies, (ii) patients diagnosed with hip fractures, and (iii) all hospital wide encounters, during the study period. Pre-AI and post-AI time periods were defined around the deployment dates of the ICH and PE modules, respectively. The reduction in LOS was 1.30 days (95% C.I. 0.1–2.5), resulting in an observed percentage decrease of 11.9% (*p* value = 0.032), for ICH and 2.07 days (95% C.I. 0.1–4.0), resulting in an observed percentage decrease of 26.3% (*p* value = 0.034), for PE when comparing the pre-AI and post-AI time periods. Reductions in LOS were observed in the ICH pre-AI and post-AI time period group for patients that were not diagnosed with ICH, but that underwent related imaging, 0.46 days (95% C.I. 0.1–0.8) resulting in an observed percentage decrease of 5% (*p* value = 0.018), and inpatients that were diagnosed with hip fractures, 0.60 days (95% C.I. 0.1–1.2) resulting in an observed percentage decrease of 8.3% (*p* value = 0.004). No other significant decrease in length of stay was observed in any of the other patient groups. The introduction of computer-aided triage and prioritization software into the radiological workflow was associated with a significant decrease in length of stay for patients diagnosed with ICH and PE.

## 1. Introduction

Radiologists face a demand for improved healthcare efficiency under a simultaneously increasing workload [1]. Artificial intelligence (AI), a popular topic within radiology over the past decade, has shown promising applications for enhancing radiologist productivity and efficiency [2, 3]. AI algorithms used for lesion detection, case triage, and workflow management can prioritize critical cases to accelerate diagnosis and reduce study turnaround time (TAT) [4]. While AI has been shown to improve radiology workflow processes, the role AI plays in other healthcare efficiency metrics, such as hospital length of stay (LOS), is unclear.

Hospital LOS is a crucial component of healthcare efficiency as it directly impacts healthcare costs. Generally, reduced LOS translates to reduced cost and substantial hospital savings [5–7]. Excessive LOS, on the other hand, leads to increased cost and potentially, clinical complications related to the increased risk of adverse events [8]. Improved radiology workflow and efficiency, including decreased study acquisition and time of interpretation, have been shown to contribute to reduced hospital LOS [9].

Our institution recently implemented AI triage software for intracranial hemorrhage (ICH) detection on CT head (CTH) and pulmonary embolism (PE) detection on the CT chest angiogram (CTCA). The purpose of our study was to determine whether there was a difference in LOS in patients diagnosed with ICH or PE before and after the implementation of this (AI) triage software.

## 2. Methods

A retrospective review was performed for patients that underwent CT imaging procedures related to ICH and PE from April 2016 to October 2019. IRB approval was granted, and the requirement for informed consent was waived.

### 2.1. AI Implementation and Adoption

Our institution implemented an AI triage software for intracranial hemorrhage (ICH) detection on CT head (CTH) and pulmonary embolism (PE) detection on a CT chest angiogram (CTCA) by Aidoc Medical Ltd (Aidoc). Aidoc provides a cloud-based AI solution for computer-aided triage and prioritization. The solution is intended to assist hospital networks and appropriately train medical specialists in workflow triage by flagging and communication of suspected positive cases of CT studies covering multiple pathologies. The solution is based on a proprietary two-stage algorithm: a region proposal stage and a false positive reduction stage. The first stage is a 3D deep convolutional neural network (CNN) that was trained on tens of thousands CTs acquired on a diverse range of CT scanners from multiple medical centers around the world. This network is trained on segmented scans and produces a 3D segmentation map. From the segmentation map, region proposals are generated and passed as the input to the second stage of the algorithm. The second stage classifies each region as positive or negative, based on features from the last layer of the first stage and traditional image processing methods. Upon detection of suspected positive findings, the solution delivers notifications directly to the radiologist workstation. The software can be connected in a variety of manners to PACS and all relevant CT studies are automatically sent for analysis with no manual trigger. The software is vendor neutral and is FDA cleared/CE marked for use on multiple scanners from multiple manufacturers.

The Aidoc solution was deployed at our institution in November 2017 with the ICH and PE modules deployed in November 2017 and December 2018, respectively. A workstation application was installed on radiologist workstations following the November 2017 deployment. The workstation application automatically launches at login with no additional user authentication or input required. Real-time prioritization alerts are provided directly in real time to the reading radiologist for potentially acute cases that require immediate attention. The alerts appear as visual pop-up notifications with an optional chime. The solution is used by over 100 radiologists across our institution. Engagement was monitored as a part of the onboarding process (duration: 2 months for each module ICH/PE) and was defined as the number of notifications reviewed by at least one radiologist. Engagement was measured to be consistently above 99% throughout the onboarding, demonstrating a successful adoption of AI alerts in the radiologist workflow.

### 2.2. ICH Patients

All patient encounters that included noncontrast head CT procedures, identified by the DICOM study descriptions, from April 2016 to April 2019 were included. All patients that were diagnosed with ICH were identified using ICD9 codes; 430, 431, 432, 852, 853, 851, and 907 (Table 1). A control group was defined as all remaining patients that underwent related imaging that were not diagnosed with ICH.

The AI solution for ICH was deployed during November of 2017. Two time periods were defined around the availability of the AI solution, pre-AI (04/01/2016–04/30/2017) and post-AI (04/01/2018–04/30/2019). A time period from 05/01/2017 to 03/31/2018 was excluded from the analysis and defined as an onboarding buffer. The justification for excluding this time period was to avoid bias due to user training and technical stabilization of the AI solution. The pre-AI and post-AI months were matched to avoid bias due to seasonal trends.

A total of 9,552 patient encounters were identified for the analysis. 1,718 patient encounters were identified as those diagnosed with ICH and were split into 923 and 795 patient encounters for the pre-AI and post-AI time periods, respectively. 13,904 patient encounters were identified for patients that underwent related imaging but that were not diagnosed with ICH and were split into 6,070 and 7,834 patient encounters for the pre-AI and post-AI time periods, respectively.

### 2.3. PE Patients

For PE, the patient encounters were identified similarly to the ICH patients as described above. All encounters that included CTCA were identified by the DICOM study descriptions. Patients diagnosed with PE were defined using the ICD9 code 415 and ICD10 code I26. A control group was defined as all remaining patients that underwent CTCA that were not diagnosed with PE. ICD10 codes were used for PE, as ICD9 codes were phased out mid-2019 at our institution (Table 1).

The PE AI solution was deployed during December of 2018 and the pre-AI (01/2018–12/2018) and post-AI (01/2019–12/2019) time periods were defined around the deployment date similarly to the ICH periods. A smaller onboarding buffer was used due to a shorter deployment process and radiologist familiarity with solution workflow.

A total of 5,254 patient encounters were identified for the analysis. 400 patient encounters were identified as diagnosed with PE and were split into 230 and 170 patient encounters for the pre-AI and post-AI time periods, respectively. 4,854 patient encounters were identified for patients who underwent related imaging that were not diagnosed with PE and were split into 2,321 and 2,533 patient encounters for the pre-AI and post-AI time periods, respectively.

### 2.4. Additional Control Groups

In addition to the relevant CT imaging control groups, two additional control groups were investigated. The first group included patients who were diagnosed with hip fractures (ICD9: 820, 821). Hip fracture was chosen as a comparison group due to acuity and treatment-related factors. Patients typically present with an acute fracture which is diagnosed clinically and radiographically. The diagnosis is followed by treatment (operative or conservative) and a hospital stay, which was found at our institution to have similar length to those for ICH and PE. The second group included all hospital-wide patient encounters at our institution. The justification to include all hospital-wide patient encounters was to exclude any trends due to shifts in general hospital policies. For both hip fractures and hospital-wide patients, the length of stay was evaluated independently twice across the matching pre-AI and post-AI time periods defined for the PE and ICH subgroups.

For the ICH time period, 3,058 hip fracture encounters were identified and were split into 1,397 and 1,661 for the pre-AI and post-AI time periods, respectively. 257,615 hospital-wide patient encounters were identified and were split into 123,782 and 133,833 for the pre-AI and post-AI, respectively.

For the PE time period, 2,940 hip fracture encounters were identified and were split into 1,530 and 1,410 for the pre-AI and post-AI time periods, respectively. 265,377 hospital-wide patient encounters were identified and split into 129,417 and 135,960 for the pre-AI and post-AI, respectively.

### 2.5. Length of Stay

All patient encounters within the identified groups were exported from the electronic health record system. The length of stay per patient encounter was defined as the time period in fraction of days between the hospital admission and the hospital discharge. All patient encounters that were longer than 120 days were excluded from the analysis as outliers.

### 2.6. Statistical Analysis

For each patient group and time period, the mean length of stay and 95% CI of the mean was calculated. The mean difference between the post-AI and pre-AI corresponding groups was calculated and a two-sided *t* test was evaluated to reject the null hypothesis.

## 3. Results

### 3.1. ICH Patients

**Figure 1:**
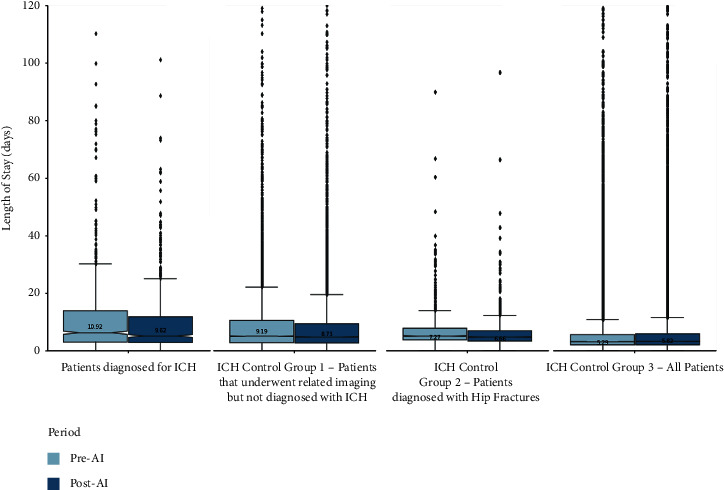
Box plots of the ICH-related patient cohorts.

For the ICH-diagnosed patients (Figure 1), a mean LOS of 10.92 and 9.62 days was observed for the pre-AI and post-AI time periods, respectively. The mean difference was 1.30 days (*p* value = 0.032), which resulted in an observed percentage decrease of 11.9%. For the group that underwent related ICH imaging but was not diagnosed with ICH, a mean LOS of 9.19 and 8.73 days was observed for the pre-AI and post-AI time periods, respectively. The mean difference was 0.46 days (*p* value = 0.018), which resulted in an observed percentage decrease of 5.0% (Table 2).

For hip fracture-diagnosed patients, a mean LOS of 7.26 and 6.66 days was observed for the pre-AI and post-AI time periods, respectively. The mean difference was 0.60 days (*p* value = 0.004), which resulted in an observed percentage decrease of 8.3%. For the hospital-wide patients, a mean LOS of 5.29 and 5.82 days was observed for the pre-AI and post-AI time periods, respectively. The mean difference was 0.54 days (*p* value < 0.001), which resulted in an observed percentage increase of 10.0% (Table 2).

### 3.2. PE Patients

**Figure 2:**
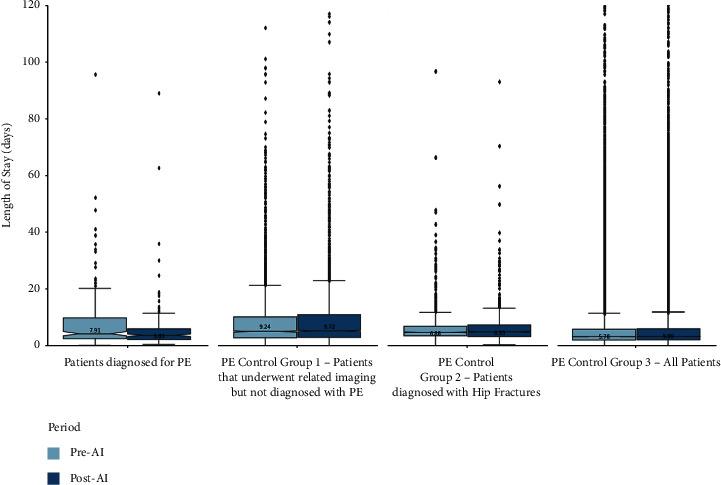
Box plots of the PE-related patient cohorts.

For the PE-diagnosed patients (Figure 2), a mean LOS of 7.91 and 5.83 days was observed for the pre-AI and post-AI time periods, respectively. The mean difference was 2.07 days (*p* value = 0.034), which resulted in an observed percentage decrease of 26.3%. For the group that underwent related PE imaging but was not diagnosed with PE, a mean LOS of 9.24 and 9.72 days was observed for the pre-AI and post-AI time periods, respectively. The mean difference of 0.49 days (*p* value = 0.157) was not statistically significant and resulted in an observed percentage increase of 5.2% (Table 3).

For hip fracture-diagnosed patients, a mean LOS of 6.90 and 6.69 days was observed for the pre-AI and post-AI time periods, respectively. The mean difference of 0.21 days (*p* value = 0.083) was not statistically significant and resulted in an observed percentage decrease of 3.0%. For the hospital-wide patients, a mean LOS of 6.51 and 6.35 days was observed for the pre-AI and post-AI time periods, respectively. The mean difference of 0.16 days (*p* value = 0.656) was also not statistically significant, resulting in an observed percentage decrease of 2.5% (Table 3).

## 4. Discussion

The purpose of the study was to determine whether there was a difference in LOS in patients diagnosed with ICH or PE prior to and following implementation of the AI-based triage software. The purpose of the software is to rapidly detect acute cases containing critical findings, such as patients with PE or ICH, and shorten the time to clinical team notification. In the absence of such a system, a first-in-first-out (FIFO) clinical workflow is used for the diagnosis and treatment of patients. Past studies have shown that reducing time to diagnosis and treatment by notifying the radiologist faster than the (FIFO) standard of care enables the clinical team to diagnose and treat the patient earlier, which is important in both PE and ICH outcomes. For example, a reduction of even two hours in the time from hospital arrival to the start of anticoagulation therapy has been shown to significantly increase PE patient survivors [10]. In the case of ICH, it has been shown that hematoma expansion occurs typically in the first few hours after bleeding starts and that hematoma volume is an important early predictor of deterioration [11]. Further studies have shown that early aggressive medical management can improve outcomes. Similar studies have been conducted with PE, demonstrating that the timely treatment of PE has been shown to reduce mortality and shorten LOS [12–14].

This study evaluated the impact of implementing an AI-based triage software into the clinical workflow by measuring LOS statistics for patient admission at our institution across two pre-AI and post-AI time periods. Statistics were compared between patients diagnosed with ICH or PE and patients not diagnosed with ICH or PE, windowed around the activation of the respective (AI) triage software. In the cohort comprising all patient encounters, no statistically significant reductions in LOS were identified when comparing the pre-AI and post-AI period. Therefore, in the time periods evaluated, LOS broadly did not change across our hospital system. In contrast, the largest significant reductions in LOS, 1.30 days (11.9%) in patients diagnosed with ICH and 2.07 days (26.3%) in patients diagnosed with PE, were observed among patients diagnosed with ICH or PE when comparing the pre-AI and post-AI time periods in each respective group. These changes in LOS in the ICH and PE cohorts from pre-AI to post-AI periods suggest a change due to the triage software implementation. Smaller magnitude statistically significant reductions in LOS were also observed in two of the control groups: (a) the ICH pre-AI and post-AI time period group for patients that were not diagnosed with ICH, but that underwent related imaging, 0.46 days (5%), and (b) in patients that were diagnosed with hip fractures, 0.60 days (8.3%). The reasons for these decreases in LOS remain unclear but may have been related to parallel LOS interventions undertaken during this time by the Department of Orthopedics (known to have occurred during the time periods studied in the case of hip fractures) as well as general efforts to decrease LOS across the hospital that were ongoing. It is notable that the absolute and percentage decreases in LOS in the ICH and PE groups were larger than in both the hip fracture and non-ICH-related imaging groups.

At our institution, the introduction of computer-aided triage and prioritization appears to have coincided with a significant decrease in length of stay for patients diagnosed with ICH and PE. The absolute and percentage decreases in LOS in these groups were also greater than the differences observed in other patient groups evaluated. In fact, during the studied time periods, several evaluated patient groups demonstrated increased LOS, supporting a unique effect in the patient groups impacted by this intervention. Further work is required to investigate if the observed link is causal. The current study does not explore if there are other reasons for the reduction, either related or unrelated to the use of AI. This remains an unanswered question and an open opportunity for future research.

One potential explanation for the observed LOS reduction in the ICH and PE groups is that by adopting an AI-based triage and prioritization system, radiologist sensitivity to previously undiagnosed small PE/ICH was augmented. Therefore, increased sensitivity may have led to an increased prevalence of short LOS encounters in patients in low-risk or low symptom categories. The severity of diagnosed PE and ICH was not evaluated in this study. However, the similarity in the total number of admissions in the pre- and post-AI periods does not appear to directly support this hypothesis.

There are some limitations of the present study. First, it would have been interesting to measure the differences by a comparison control study within the same time period, where half of the studies got triage and half did not. This would have allowed a more direct comparison over these two groups over time. Unfortunately, such approaches are not necessarily practical and can introduce ethical questions concerning patient care. Also, this was an observational study with direct measurement of individual radiologist engagement with the software. Lastly, we do not address whether the length of stay was associated with equivalent outcomes. Further investigation is warranted.

In addition to purely quantitative metrics and clinical outcomes, the introduction of AI-based triage to the clinical workflow at our institution raises a number of complex medicolegal and ethical questions whose full discussion is beyond the scope of this investigation but worth mentioning when AI is concerned. In the context of emergent findings such as PE and ICH, the clinical workflow is complex and timing is critical, so it is impossible for a patient to be aware that an AI tool has been utilized in their care. To date, the solutions used at our institution have been approved for and are intended for triage usage only, but the consideration of usage as a diagnostic tool is important to consider in the future. This presents unique challenges to both the radiologists and downstream clinicians, as discrepancies between radiologists and AI interpretation are not uncommon, leading to a question over which is correct. The somewhat opaque “black box” nature of the software at our institution as well as AI products in general confounds the issue, as the verification of the process behind findings is out of the scope of most radiologists [7].

## 5. Conclusion

In conclusion, since hospital utilization is a massive driver of healthcare system costs and there has been a large push to drive down hospital length of stay over a wide variety of conditions, any avenue towards decreasing length of stay could be beneficial for healthcare systems. The advent of AI triage software offers a new path towards such reductions and we demonstrate an association between the introduction of triage software and decreased hospital LOS.

## Figures and Tables

**Table 1:** List of ICD codes used and the related pathologies.

Code	Description	Pathology
ICD9\430	Subarachnoid hemorrhage	ICH
ICD9\431	Intracerebral hemorrhage	ICH
ICD9\432	Other and unspecified intracranial hemorrhage	ICH
ICD9\851	Cerebral laceration and contusion	ICH
ICD9\852	Subarachnoid subdural and extradural hemorrhage following an injury	ICH
ICD9\853	Other and unspecified intracranial hemorrhage following an injury	ICH
ICD9\907	Late effects of injuries to the nervous system	ICH
ICD9\415	Acute pulmonary heart disease	PE
ICD10\I26	Pulmonary embolism	PE
ICD9\820	Fracture of the neck of the femur	Hip fractures
ICD9\821	Fracture of other and unspecified parts of the femur	Hip fractures

ICD : international classification of diseases. PE : pulmonary embolism. ICH : intracranial hemorrhage.

**Table 2:** Statistical descriptions of the ICH-related patient cohorts.

	Pre-AI	Post-AI
*Patients diagnosed for ICH*		
Mean (CI 95%)	10.92 (10.05–11.79)	9.62 (8.81–10.43)
Difference in means	1.30 (*p* value = 0.032)	
% Difference in means	11.9%	
Median	6.25	5.12
Standard deviation	13.48	11.62
25th percentile	3.00	2.88
75th percentile	13.88	11.77
99th percentile	75.99	55.97

*ICH control group 1—patients that underwent related imaging but were not diagnosed with ICH*		
Mean (CI 95%)	9.19 (8.90–9.47)	8.73 (8.47–8.98)
Difference in means	0.46 (*p* value = 0.018)	
% Difference in means	5.0	
Median	5.04	4.79
Standard deviation	12.05	12.00
25th percentile	2.79	2.71
75th percentile	10.54	9.38
99th percentile	64.8	63.0

*ICH control group 2—patients diagnosed with hip fractures*		
Mean (CI 95%)	7.26 (6.86–7.66)	6.66 (6.27–7.04)
Difference in means	0.60 days (*p* value = 0.004)	
% Difference in means	8.3%	
Median	5.08	4.75
Standard deviation	7.66	7.91
25th percentile	3.79	3.38
75th percentile	7.83	6.92
99th percentile	35.08	34.29

*ICH control group 3—all patients*		
Mean (CI 95%)	5.29 (5.24–5.33)	5.82 (5.77–5.87)
Difference in means	−0.54 days (*p* value < 0.001)	
% Difference in means	−10%	
Median	3.17	3.21
Standard deviation	7.47	9.11
25th percentile	2.04	2.04
75th percentile	5.59	5.88
99th percentile	37.59	48.75

ICD : international classification of diseases. PE : pulmonary embolism. ICH : intracranial hemorrhage. AI : artificial intelligence. CI 95%: 95% confidence interval.

**Table 3:** Statistical descriptions of the PE-related patient cohorts.

	Pre-AI	Post-AI
*Patients diagnosed for PE*		
Mean (CI 95%)	7.91 (6.58–9.23)	5.83 (4.44 –7.23)
Difference in means	2.07 days (*p* value = 0.034)	
% Difference in means	26.3%	
Median	4.27	3.73
Standard deviation	10.21	9.22
25th percentile	2.49	2.17
75th percentile	9.85	5.99
99th percentile	45.86	44.34

*PE control group 1—patients that underwent related imaging but were not diagnosed with PE*		
Mean (CI 95%)	9.24 (8.77–9.70)	9.72 (9.23 –10.21)
Difference in means	−0.49 days (*p* value = 0.157)	
% Difference in means	−5.2%	
Median	5.04	5.25
Standard deviation	11.90	13.0
25th percentile	2.83	2.96
75th percentile	10.21	10.96
99th percentile	62.00	70.04

*PE control group 2—patients diagnosed with hip fractures*		
Mean (CI 95%)	6.88 (6.45–7.30)	6.93 (6.53–7.33)
Difference in means	0.21 days (*p* value = 0.083)	
% Difference in means	3.0%	
Median	4.77	4.92
Standard deviation	8.46	7.59
25th percentile	3.54	3.25
75th percentile	6.88	7.38
99th percentile	42.28	37.17

*PE control group 3—all patients*		
Mean (CI 95%)	6.51 (6.42–6.60)	6.35 (6.28–6.41)
Difference in means	0.16 days (*p* value = 0.656)	
% Difference in means	2.5%	
Median	3.21	3.25
Standard deviation	16.15	12.53
25th percentile	2.04	2.08
75th percentile	5.83	6.00
99th percentile	60.15	61.04

ICD : international classification of diseases. PE : pulmonary embolism. ICH : intracranial hemorrhage. AI : artificial intelligence. CI 95%: 95% confidence interval.

## Data Availability

The data used to support the findings of this study are restricted by the Cedars-Sinai Medical Center IRB in order to protect patient privacy. Data are available from the corresponding author at charlotte.lansky@cshs.org for researchers who meet the criteria to access confidential data.
